# Supplementary material for: Hybrid Models and Biological Model Reduction with PyDSTool
Source: PLoS Comput Biol. 2012 Aug 9;8(8):e1002628. doi: 10.1371/journal.pcbi.1002628 (PMC3415397; doi:10.1371/journal.pcbi.1002628)
Supplement: Text S4 — Complete source code for the PyDSTool package (version 0.88.120504). Includes API documentation and help files linking to web pages. This file is identical to the current public release on Sourceforge.net. (ZIP) [file pcbi.1002628.s004.zip › PyDSTool/html/PyDSTool.Toolbox.data_analysis-module.html]

xml version="1.0" encoding="ascii"?


PyDSTool.Toolbox.data\_analysis


| Home | Trees | Indices | Help | PyDSTool |
| --- | --- | --- | --- | --- |

Package PyDSTool ::
Package Toolbox ::
Module data\_analysis

[hide private]

[frames] | no frames]

# Module data\_analysis

source code

Data analysis utilities

Robert Clewley, August 2006


|  |  |
| --- | --- |
| Classes | [hide private] |

data\_bins  
Class for data binning.


|  |  |
| --- | --- |
| Functions | [hide private] |

|  |  |
| --- | --- |
| lfilter(b, a, x, axis=-1, zi=None)  Filter data along one-dimension with an IIR or FIR filter. |  |

|  |  |
| --- | --- |
| butter(N, Wn, btype=`'``low``'`, analog=0, output=`'``ba``'`)  Butterworth digital and analog filter design. |  |

|  |  |
| --- | --- |
| PCANode(\*args, \*\*kwargs) | source code |

|  |  |
| --- | --- |
| lfilter\_zi(b, a) | source code |

|  |  |
| --- | --- |
| filtfilt(b, a, x) | source code |

|  |  |
| --- | --- |
| doPCA(in\_pts, D, tol=0.8) | source code |

|  |  |
| --- | --- |
| get\_evec(p, dim) | source code |

|  |  |
| --- | --- |
| pca\_dim(pca, covering, data, refpt, tol=0.8) | source code |

|  |  |
| --- | --- |
| get\_residual(pcanode, pts) | source code |

|  |  |
| --- | --- |
| plot\_PCA\_residuals(data, D=None, newfig=True, marker=`'``o``'`, silent=False) | source code |

|  |  |
| --- | --- |
| plot\_PCA\_spectrum(data, D) | source code |

|  |  |
| --- | --- |
| log\_distances(m, sampleix=0, doplot=True, quiet=True, logv=None, plotstyle=None, return\_ixs=False)  Log distances (L2-norm) of data points from a reference point (sampleix). | source code |

|  |  |
| --- | --- |
| log\_distances\_with\_D(m, sampleix, logv=None, return\_ixs=False)  Log distances (L2-norm) of data points from a reference point (sampleix). | source code |

|  |  |
| --- | --- |
| rectify(xa, full=False)  Half-wave (default) or full-wave signal rectification: specify using optional full Boolean flag. | source code |

|  |  |
| --- | --- |
| get\_linear\_regression\_residual(pfit, x, y, weight=`'``'`, w=0)  Also see scipy.stat.linregress for a more sophisticated version | source code |

|  |  |
| --- | --- |
| fitline(x, y, lo=0, hi=1, doplot=True, quiet=True, linewidth=2)  fitline takes the position of low and high fit limits and returns the slope. | source code |

|  |  |
| --- | --- |
| find\_closest\_val(x, a, eps=1e-07, which\_norm=2)  Find one value in a closer than eps to x | source code |

|  |  |
| --- | --- |
| find\_nearby\_ball(data, refix, r, which\_norm=2, include\_ref=False)  Return indices of all points in data that are inside a ball of radius r from the reference point (not included). | source code |

|  |  |
| --- | --- |
| find\_nearby\_annulus(data, refix, rlo, rhi, which\_norm=2)  Return a list containing input data's indices of all neighbours of the reference point within the range of distances d such that dlo < d < dhi | source code |

|  |  |
| --- | --- |
| find\_central\_point(data, which\_norm=2)  Find the index of a point closest to the 'centre' of a data set. | source code |

|  |  |
| --- | --- |
| find\_recurrences(data, refix, r, times=None, ignore\_small=0, which\_norm=2)  Find recurrences of a trajectory in a ball of radius r centred at the reference point given by refix in data. | source code |

|  |  |
| --- | --- |
| find\_diameter(data, eps, which\_norm=2)  Find approximate diameter of data set, up to tolerance defined by eps > 0. | source code |

|  |  |
| --- | --- |
| dist\_between\_datasets(data1, data2, which\_norm=2) | source code |

|  |  |
| --- | --- |
| out\_of\_seq(a, inc\_only=False)  Determine if and where an integer array a is not increasing or decreasing in sequence by 1. | source code |

|  |  |
| --- | --- |
| whiten(data)  Subtract mean and divide by standard deviation in each column of data | source code |

|  |  |
| --- | --- |
| find\_knees(data, tol=1.0, inlog=False, verbose=False)  Find one or more 'knees' in data, according to high second derivatives. | source code |

|  |  |
| --- | --- |
| recurrence\_times(ts, partitions)  Internal function for use by find\_recurrences | source code |

|  |  |
| --- | --- |
| find\_from\_sorted(x, v, next\_largest=1, indices=None) | source code |

|  |  |
| --- | --- |
| colormap(mag, cmin, cmax)  Return a tuple of floats between 0 and 1 for the red, green and blue amplitudes. | source code |

|  |  |
| --- | --- |
| dump\_progress(x, tot, filename=`'``progress_info.dat``'`)  Send progress report to a file | source code |

|  |  |
| --- | --- |
| second\_diff(data, i)  Naive second difference formula at position i of data | source code |

|  |  |
| --- | --- |
| \_adjust\_ix(i, n)  Internal helper function | source code |


|  |  |
| --- | --- |
| Variables | [hide private] |

\_num\_types = `(<type 'float'>, <type 'int'>, <type 'numpy.float...`

\_int\_types = `(<type 'int'>, <type 'numpy.integer'>)`

\_seq\_types = `(<type 'list'>, <type 'tuple'>, <type 'numpy.ndar...`

newaxis = `None`

r\_ = `<numpy.lib.index_tricks.RClass object at 0x11542f0>`

\_PCA\_utils = `['doPCA', 'get_evec', 'pca_dim', 'get_residual', ...`

\_metric\_utils = `['log_distances', 'log_distances_with_D']`

\_analysis\_utils = `['find_knees', 'whiten', 'find_closest_val',...`

\_fit\_utils = `['get_linear_regression_residual', 'fitline']`

\_filter\_utils = `['lfilter_zi', 'filtfilt', 'lfilter', 'butter'...`

dict\_tol = `array([ 1.00000000e-08])`


|  |  |
| --- | --- |
| Function Details | [hide private] |

|  |  |
| --- | --- |
| lfilter(b, a, x, axis=-1, zi=None) |  |

```
Filter data along one-dimension with an IIR or FIR filter.

Description

  Filter a data sequence, x, using a digital filter.  This works for many
  fundamental data types (including Object type).  The filter is a direct
  form II transposed implementation of the standard difference equation
  (see "Algorithm").

Inputs:

  b -- The numerator coefficient vector in a 1-D sequence.
  a -- The denominator coefficient vector in a 1-D sequence.  If a[0]
       is not 1, then both a and b are normalized by a[0].
  x -- An N-dimensional input array.
  axis -- The axis of the input data array along which to apply the
          linear filter. The filter is applied to each subarray along
          this axis (*Default* = -1)
  zi -- Initial conditions for the filter delays.  It is a vector
        (or array of vectors for an N-dimensional input) of length
        max(len(a),len(b)).  If zi=None or is not given then initial
        rest is assumed.  SEE signal.lfiltic for more information.

Outputs: (y, {zf})

  y -- The output of the digital filter.
  zf -- If zi is None, this is not returned, otherwise, zf holds the
        final filter delay values.

Algorithm:

  The filter function is implemented as a direct II transposed structure.
  This means that the filter implements

  a[0]*y[n] = b[0]*x[n] + b[1]*x[n-1] + ... + b[nb]*x[n-nb]
                        - a[1]*y[n-1] - ... - a[na]*y[n-na]

  using the following difference equations:

  y[m] = b[0]*x[m] + z[0,m-1]
  z[0,m] = b[1]*x[m] + z[1,m-1] - a[1]*y[m]
  ...
  z[n-3,m] = b[n-2]*x[m] + z[n-2,m-1] - a[n-2]*y[m]
  z[n-2,m] = b[n-1]*x[m] - a[n-1]*y[m]

  where m is the output sample number and n=max(len(a),len(b)) is the
  model order.

  The rational transfer function describing this filter in the
  z-transform domain is
                              -1               -nb
                  b[0] + b[1]z  + ... + b[nb] z
          Y(z) = ---------------------------------- X(z)
                              -1               -na
                  a[0] + a[1]z  + ... + a[na] z
```

|  |  |
| --- | --- |
| butter(N, Wn, btype=`'``low``'`, analog=0, output=`'``ba``'`) |  |

```
Butterworth digital and analog filter design.

Description:

  Design an Nth order lowpass digital or analog Butterworth filter
  and return the filter coefficients in (B,A) or (Z,P,K) form.

See also buttord.
```

|  |  |
| --- | --- |
| log\_distances(m, sampleix=0, doplot=True, quiet=True, logv=None, plotstyle=None, return\_ixs=False) | source code |

Log distances (L2-norm) of data points from a reference point
(sampleix).

For multiple calls to this function on the same data, it's faster to
pass logv precomputed.

return\_ixs=True also returns the point indices from the sort to be
returned in this argument in a third return value.

|  |  |
| --- | --- |
| log\_distances\_with\_D(m, sampleix, logv=None, return\_ixs=False) | source code |

Log distances (L2-norm) of data points from a reference point
(sampleix). Additionally, it returns a list of the inter-point distances,
relative to the reference point, and the inverse of this mapping.

For multiple calls to this function on the same data, it's faster to
pass logv precomputed.

return\_ixs=True returns the point indices from the sort in a fifth
return value. This identifies the points in the returned list of
inter-point distances and its inverse.

|  |  |
| --- | --- |
| rectify(xa, full=False) | source code |

Half-wave (default) or full-wave signal rectification: specify using
optional full Boolean flag. xa must be an array

|  |  |
| --- | --- |
| fitline(x, y, lo=0, hi=1, doplot=True, quiet=True, linewidth=2) | source code |

fitline takes the position of low and high fit limits and returns the
slope. Integer indices in the x data set can be specified for the low and
high limits, otherwise use a fraction between 0 and 1.

In application to fractal dimension estimation, if x = log d (radius,
a.k.a. inter-point distance) and y = log v (index, a.k.a. estimate of
volume at a given radius) then the slope is the dimension estimate of the
data set.

Also see scipy.stat.linregress for a more sophisticated version

|  |  |
| --- | --- |
| find\_central\_point(data, which\_norm=2) | source code |

Find the index of a point closest to the 'centre' of a data set. The
centre is defined to be where the mean of the points' position vectors
relative to the centre is minimized in each direction.

|  |  |
| --- | --- |
| find\_recurrences(data, refix, r, times=None, ignore\_small=0, which\_norm=2) | source code |

```
Find recurrences of a trajectory in a ball of radius r centred at the
reference point given by refix in data.

If recurrence times are desired, pass an associated time array corresponding
to the points in the data.

If ignore_small > 0, recurrences of fewer than this number of data points
   will be treated as anomalous and ignored completely.

Returns a structure (class common.args) having attributes:
    partitions -- pairs of entry and exit indices for each recurrence in
            the ball
    ball_ixs -- all consecutive indices into data for each recurrence
    partitions_lengths -- number of indices in each partition
    rec_times -- times to next recurrence in the set from previous
```

|  |  |
| --- | --- |
| find\_diameter(data, eps, which\_norm=2) | source code |

Find approximate diameter of data set, up to tolerance defined by eps
> 0.

Data assumed to be a rank-2 array.

|  |  |
| --- | --- |
| find\_knees(data, tol=1.0, inlog=False, verbose=False) | source code |

Find one or more 'knees' in data, according to high second
derivatives.

inlog option finds knees in the logs of the data entries. tol=1. works
well if inlog=False tol=0.3 works well if inlog=True

|  |  |
| --- | --- |
| colormap(mag, cmin, cmax) | source code |

Return a tuple of floats between 0 and 1 for the red, green and blue
amplitudes. Function originally named floatRgb and written by Alexander
Pletzer, from the Python Cookbook.

  


|  |  |
| --- | --- |
| Variables Details | [hide private] |

### \_num\_types

Value:
:   ```
    (<type 'float'>,
     <type 'int'>,
     <type 'numpy.floating'>,
     <type 'numpy.integer'>)
    ```

### \_seq\_types

Value:
:   ```
    (<type 'list'>, <type 'tuple'>, <type 'numpy.ndarray'>)
    ```

### \_PCA\_utils

Value:
:   ```
    ['doPCA',
     'get_evec',
     'pca_dim',
     'get_residual',
     'plot_PCA_residuals',
     'plot_PCA_spectrum']
    ```

### \_analysis\_utils

Value:
:   ```
    ['find_knees',
     'whiten',
     'find_closest_val',
     'out_of_seq',
     'find_nearby_ball',
     'find_nearby_annulus',
     'data_bins',
     'find_central_point',
    ...
    ```

### \_filter\_utils

Value:
:   ```
    ['lfilter_zi', 'filtfilt', 'lfilter', 'butter', 'rectify']
    ```

  


| Home | Trees | Indices | Help | PyDSTool |
| --- | --- | --- | --- | --- |

|  |  |
| --- | --- |
| Generated by Epydoc 3.0.1 on Fri May 4 15:24:04 2012 | http://epydoc.sourceforge.net |
